# PKMζ-KIBRA interactions, molecular turnover, and memory

**DOI:** 10.1186/s13041-026-01284-4

**Published:** 2026-03-12

**Authors:** Changchi Hsieh, David A. Cano, Panayiotis Tsokas, James E. Cottrell, André Antonio Fenton, Harel Shouval, Todd Charlton Sacktor

**Affiliations:** 1 Department of Physiology and Pharmacology, The Robert F. Furchgott Center for Neural and Behavioral Science, State University of New York Downstate Health Sciences University, Brooklyn, NY 11203 USA; 2 College of Medicine, State University of New York Downstate Health Sciences University, Brooklyn, NY 11203 USA; 3 Department of Anesthesiology, State University of New York Downstate Health Sciences University, Brooklyn, NY 11203 USA; 4 Department of Pathology, State University of New York Downstate Health Sciences University, Brooklyn, NY 11203 USA; 5 Center for Neural Science, New York University, New York, NY 10003 USA; 6 Neuroscience Institute at NYU Langone Medical Center, New York, NY 10016 USA; 7 Department of Neurobiology and Anatomy, University of Texas Medical at Houston, Houston, TX 77030 USA; 8 Department of Neurology, State University of New York Downstate Health Sciences University, Brooklyn, NY 11203 USA

**Keywords:** PKMzeta, PKM-zeta, long-term potentiation (LTP), WWC1

## Abstract

How can the molecules that strengthen synaptic connections maintain memory in the face of molecular turnover? Our previous work showed that persistent interaction between the postsynaptic scaffolding protein, KIBRA, and the autonomously active PKC isoform, PKMζ, is crucial for maintaining synaptic long-term potentiation (LTP) and memory lasting at least a month. This duration is longer than the lifespans of individual KIBRA and PKMζ molecules. Biophysical modeling of the interaction suggests oligomers of KIBRA-PKMζ dimers, but not individual dimers or monomers, can overcome molecular turnover by continuously incorporating newly synthesized KIBRA and PKMζ, replacing those that have degraded. Here we used AlphaFold 3 to predict the structures of KIBRA-PKMζ heterodimers and heterohexamers and to examine the sites of action of two different inhibitors of KIBRA-PKMζ interaction that disrupt established late-LTP and long-term memory. The structures predict that the peptide K-ZAP blocks formation of heterodimers, whereas the small molecule ζ-stat prevents PKMζ of one heterodimer from binding a second KIBRA and PKMζ, essential for forming larger oligomeric structures. We show that ζ-stat, like K-ZAP, disrupts 1-month-old spatial memory. Thus, continuous formation of KIBRA-PKMζ oligomers can be a core molecular mechanism driving the persistence of long-term memory in the face of molecular turnover.

## Introduction

How can memories be maintained when all the molecular components of synapses are continually replaced [1]? Autonomously active protein kinases can maintain synaptic potentiation, but they turn over within hours to days [2]. Crick and Lisman independently proposed a mechanism to prolong the action of these kinases for the lifespan of a memory— although the molecules themselves are not long-lived, the interactions between them can persist while newly synthesized proteins continually replace those that degrade [1, 2].

Two kinases that potentiate synaptic transmission become autonomously active [3]. One is Ca^2+^/calmodulin-dependent kinase II (CaMKII) that becomes Ca^2+^/calmodulin-independent through autophosphorylation and is crucial for the initial stages of inducing LTP and memory [4]. The other is the continuously active atypical PKC, PKMζ, that is critical for sustaining the mechanistically distinct, enduring maintenance of wild-type LTP and long-term memory [5]. Once translated [6], the steady-state increase in PKMζ persists for hours to maintain late-LTP in hippocampal slices and for at least a month during spatial memory in specific synaptodendritic regions of the hippocampal neurons that were active during learning [7, 8].

**Fig. 1:**
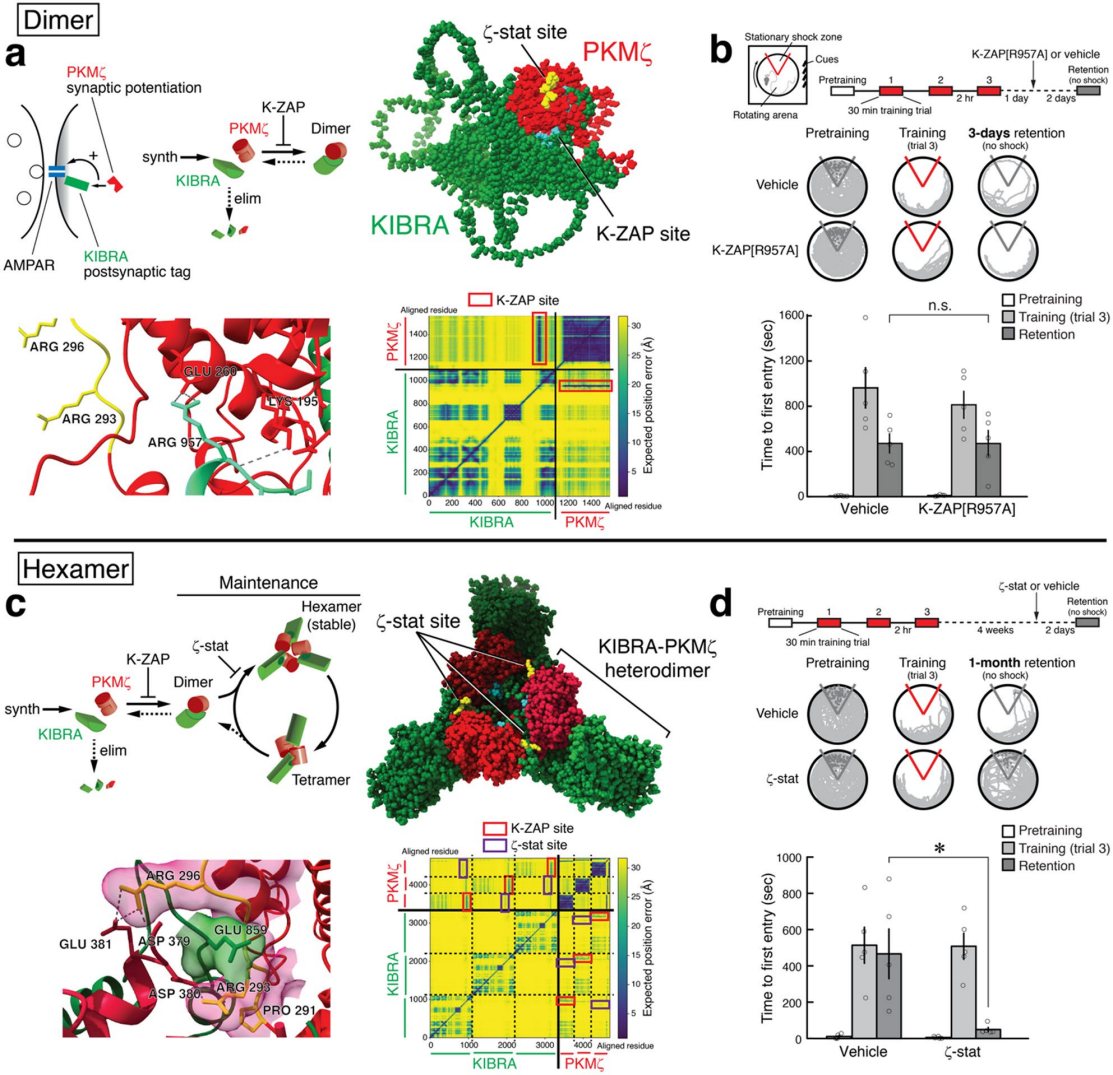
KIBRA-PKMζ interactions predicted to form dimers and hexamers maintain long-term spatial memory. **a** Above left, schematic showing elements of KIBRA-PKMζ synaptic potentiation. Postsynaptic KIBRA acts as a synaptic tag, which targets PKMζ that potentiates AMPAR responses. Above middle, summary of kinetic model of formation of KIBRA-PKMζ heterodimers from new synthesis (synth) of monomers. High levels of KIBRA-tags (green) and PKMζ (red) cannot be maintained because the dimers dissociate and elimination (elim) of monomers is rapid. Above right, heterodimer structure predicted by AlphaFold 3. KIBRA’s K-ZAP sequence (blue) interacts with PKMζ, whereas the PKMζ handle does not interact with KIBRA (ζ-stat binding site, i.e., 7 amino acids from P291 to F297, shown in yellow). Below left, the 3 hydrogen bonds between K-ZAP arginine-957 and PKMζ are shown; there are 20 hydrogen bonds between the total K-ZAP sequence and PKMζ. The arginines in the ζ-stat-binding motif of PKMζ do not interact with KIBRA (yellow, amino-acid numbering based on PKMζ sequence [6]). Below right, predicted aligned error plot of the KIBRA-PKMζ heterodimer. K-ZAP sequence interactions with PKMζ are shown in red boxes. **b** Whereas K-ZAP disrupts 1-day- and 1-month-old spatial memory [10] (shown in kinetic model, **a** above middle), bilateral hippocampal injections of inactive, mutated K-ZAP peptide (myristoyl-FVRNSLEARSVRMKRPS, 5 nmol in 0.5 µl vehicle per side) do not disrupt 1-day-old memory. Above left, schematic of active place avoidance training apparatus with a slowly rotating arena containing a nonrotating shock zone sector (shown in red). Visual cues located on the walls of the room are needed to avoid the shock zone. Above right, protocol for active place avoidance. Three 30-min training sessions (red) are separated by 2 h; injections are 1 day later. We tested memory retention without shock 2 days after the injection [10]. In active place avoidance, comparisons are between the pair of acquisition tests and between the pair of retention tests of each experiment [10]. With each experimental comparison pair, all known factors that can affect the outcome are counterbalanced between the groups by the experimental design, including time of day, season, litter, training order, and housing stress, which may account for differences in training efficacy such as between **b** and **d**. Middle, representative paths during 10 min of pretraining, at end of training trial 3 (shock zone shown in red), and 1-day memory retention. Shock zone with shock off is shown in gray. Below, mean ± SEM. ANOVA with repeated measurements reveals a single significant effect of training (pretraining, training, and retention; *F*_2,16_ = 33.81, *P* < 0.00001, *η*^2^_p_ = 0.81), and no treatment effect (vehicle and mutated K-ZAP[R957A]) nor their interaction. Bonferroni-corrected comparisons confirm that the memory retention after K-ZAP[R957A] injection is not significantly different from vehicle control (n.s., *P* = 1, n’s = 5; as initial experiments with K-ZAP[R957A] showed normal memory, for comparison with K-ZAP[R957A], vehicle controls were pooled with 4 randomly selected vehicle controls from K-ZAP experiments [10]). **c** Above left, kinetic model showing self-perpetuating formation of stable KIBRA-PKMζ hexamers in LTP/memory maintenance. Above right, predicted hexamer structure. KIBRA’s K-ZAP sequence interacts with PKMζ forming pairs, and PKMζ’s handle links the pair to a second KIBRA (dark green) and PKMζ (dark red). Below left, PKMζ’s ζ-stat binding site interacts with a second KIBRA (interaction shown with molecular surfaces) and forms 4 hydrogen bonds with a second PKMζ. Below right, predicted aligned error plot of KIBRA-PKMζ hexamers. K-ZAP sequence interaction sites with PKMζ are shown in red boxes, ζ-stat sites in purple. **d** Bilateral hippocampal injections of ζ-stat (5 nmol in 0.5 µl vehicle per side) disrupt 4-week-old spatial memory. Above, protocol for active place avoidance. Middle, representative paths during 10 min of pretraining, at end of training trial 3, and 4-week memory retention. Below, mean ± SEM. ANOVA with repeated measurements finds significant effects of training (pretraining, training, and retention; *F*_2,62_ = 25.93, *P* = 0.00001, *η*^2^_p_ = 0.76) and interaction between effects of training and treatment (vehicle and ζ-stat) (training X treatment: *F*_2,16_ = 5.79, *P* = 0.01, *η*^2^_p_ = 0.42). The 1-month memory retention was abolished by ζ-stat injected 2 days prior to the test, compared with vehicle (*, significant Tukey *post-hoc* tests, *P* = 0.008, n’s = 5). Methods. FASTA protein sequences for KIBRA (Q5SXA9) and PKMζ (Q02956-2) from *Mus musculus*, were taken from Uniprot and analyzed by AlphaFold 3 to generate protein complexes in silico. All models contained one ATP and two Mg^2+^ ions per PKMζ molecule, as well as activating post-translational modifications for PKMζ at P-threonine-227 and P-threonine-377 (corresponding to P-threonine 410 and P-threonine 560 in PKCζ). The highest confidence AlphaFold 3 output files were visualized in UCSF ChimeraX (v1.9). Hydrogen bonds were calculated by ChimeraX with distance tolerance set to 0.4 Å and angle tolerance set to 20°. Active place avoidance and intrahippocampal injections were performed as previously described [10]

To maintain LTP and memory PKMζ must continuously interact with a synaptic tag, the postsynaptic scaffolding protein KIBRA (aka WWC1), which is genetically linked to human memory performance and Alzheimer’s disease [9, 10]. After initial synthesis, KIBRA and PKMζ form complexes that persist during maintenance and continually target the kinase’s action to active synapses [10] (Fig. 1a). Importantly, two structurally distinct inhibitors disrupt KIBRA-PKMζ interaction and reverse established late-LTP and long-term spatial memory [10]. One, the peptide inhibitor K-ZAP mimics and occludes the action of a sequence in KIBRA’s C-terminus where PKMζ binds [11]. The other, the small molecule ζ-stat blocks the “handle” motif of PKMζ’s catalytic domain where KIBRA binds [10, 12].

It is crucial that PKMζ and KIBRA interact; however, biophysical modeling suggests that simple PKMζ-KIBRA heterodimers cannot permanently store information at synapses [13]. PKMζ rapidly degrades as a monomer and becomes stable on binding KIBRA [12–14]. Nevertheless, heterodimers formed after LTP induction or learning will eventually dissociate into rapidly degrading monomers. Consequently, the information they once encoded is lost.

Our biophysical model predicts that hexameric or larger oligomers composed of KIBRA-PKMζ pairs are better suited to store information over long time periods because they can survive molecular turnover [13]. Specifically, the individual molecules of a larger oligomer that degrade could be replaced because the remaining complex can serve as a template to bind newly synthesized KIBRA and PKMζ. This view predicts that the inhibitors K-ZAP or ζ-stat that disrupt KIBRA-PKMζ interactions would prevent the continual replenishment of the oligomers, and the loss of the complexes would reverse synapses from a stable potentiated to stable unpotentiated state. This outcome would permanently disrupt established late-LTP and long-term memory maintenance, a result that has been observed [10].

## Results

For heterodimers to form larger oligomers, one molecule of a species should bind to more than one molecule in the complex. To investigate if KIBRA or PKMζ have this property, we used AlphaFold 3 to predict both their dimeric and hexameric forms.

### The K-ZAP sequence of KIBRA that binds PKMζ forms KIBRA-PKMζ dimers

AlphaFold 3 predicts that heterodimers are formed by interaction between the K-ZAP sequence of KIBRA (FVRNSLERRSVRMKRPS-966), and the surface of PKMζ (Fig. 1a). However, the PKMζ-handle, where ζ-stat binds, does not appear to interact with KIBRA in the dimeric complex.

The peptide K-ZAP disrupts 1-day- and 1-month-old memory [10]. To further test whether K-ZAP interaction is critical for maintaining memory, we trained mice on an active place avoidance memory task and 1 day later bilaterally injected in hippocampus a mutated form of K-ZAP, in which the critical KIBRA arginine-957 is changed to alanine to decrease the peptide’s interaction with PKMζ [14] (Fig. 1b). In the predicted heterodimers, arginine-957 has 3 hydrogen bonds with PKMζ, and the mutation to alanine (K-ZAP[R957A]) has only 1. If K-ZAP prevents memory maintenance by interfering with PKMζ-KIBRA dimerization, then the mutated version should have no effect. As predicted, hippocampal injections of the mutated peptide K-ZAP[R957A] did not affect long-term memory retention.

*The PKMζ-handle with the* *ζ-stat binding site interacts with a second KIBRA and PKMζ* *in KIBRA-PKMζ* *hexamers.*

In heterohexamers, KIBRA’s K-ZAP sequence preserves KIBRA-PKMζ pairing, and the PKMζ-handle binds to a second KIBRA as well as a second PKMζ, linking the pairs (Fig. 1c). Two amino acids in the PKMζ handle, proline-291 and phenylalanine-297, are critical for both strong binding of PKMζ to KIBRA and the inhibitory action of ζ-stat [10]. These amino acids flank two arginines predicted to interact with a disordered region of KIBRA and the surface of another PKMζ. If these flanking amino acids are changed to the analogous amino acids of the other atypical PKCι/λ, which binds only weakly to KIBRA, the mutated PKMζ[PKCι/λ-P291Q; F297S] also binds weakly to KIBRA [10]. On changing the flanking amino acids in the structural model, the predicted number of hydrogen bonds linking the PKMζs decreases from 4 to 0 [10]. Consequently, AlphaFold predicts that the ζ-stat-binding site is key to forming and maintaining KIBRA-PKMζ hexamers. The site is precisely where the KIBRA-PKMζ pairs interact with each other. This contrasts with dimers in which the ζ-stat-binding site does not participate.

The estimated lifespans of PKMζ and KIBRA molecules in vivo are a few days [10]. We found that injecting ζ-stat to inhibit the predicted KIBRA-PKMζ oligomer-interaction site disrupts a 4-week-old spatial memory (Fig. 1d). Thus, 1-month long-term memory depends on KIBRA-PKMζ oligomers for its maintenance.

## Discussion

Biophysical modeling of KIBRA-PKMζ interactions predicted that hetero-oligomers could maintain high levels of the KIBRA-tag and PKMζ at active synapses to sustain potentiation despite protein turnover [13]. Therefore, if ζ-stat specifically prevents oligomer formation as AlphaFold predicts (Fig. 1c), then, like K-ZAP that blocks dimer formation [10], ζ-stat should disrupt memories that are maintained longer than the lifespans of individual PKMζ and KIBRA molecules. This was the case (Fig. 1d). The effect of ζ-stat is selective to PKMζ because the drug has no effect on LTP or memory maintenance in the absence of the kinase in compensated PKMζ-knockout mice, in which these long-term processes are sustained by PKCι/λ [10]. Off-target effects would not be expected to be eliminated in the knockout mice. In addition, the drug has no effect on KIBRA interactions with PKCι/λ or any other PKC isoform or CaMKIIα [10].

To determine ζ-stat’s effect on memory maintenance, we tested 1-month memory retention 2 days after ζ-stat injection when the drug is eliminated [10]. As ζ-stat disrupted memory retention even after it had dissipated, the drug has a persistent effect on 1-month memory maintenance and not just a transient effect on retrieval. The rate of oligomer degradation by ζ-stat, however, may be faster than 2 days. The rate may best be estimated by the kinetics of late-LTP reversal, as measured in hippocampal slice experiments in which the perfusion rate and concentration of drug can be controlled [10]. After initiation of drug perfusion, ζ-stat reverses late-LTP in ~ 3–4 h. Likewise, while ζ-stat disrupts memory maintenance at 3 days [10] and 1 month (Fig. 1d) post-training, the KIBRA-PKMζ complexes may have formed earlier. Again, our best estimate of the formation of oligomers from dimers is from LTP experiments that show ζ-stat blocks the development of late-LTP within ~ 2–3 h post-tetanization, indicating that functional oligomers begin forming at this time [10].

A critical feature of our kinetic model of maintenance is that the formation of hexamers from dimers should include a cooperative step [13]. The binding of PKMζ to two KIBRAs and a second PKMζ within a hexamer might provide the necessary nonlinearity (Fig. 1c). Our simple model does not exclude the possibility that other molecules are components of KIBRA-PKMζ complexes, such as PICK1 that interacts with both KIBRA and PKMζ [5, 15]. Characterizing the self-perpetuation of KIBRA-PKMζ complexes with their associated proteins might elucidate the fundamental properties of a synaptic “mnemosome” that stores information in the brain and is disrupted in disorders of memory.

## Data Availability

All data generated or analysed during this study are included in this published article.

## Abbreviations

AMPAR: α-amino-3-hydroxy-5-methyl-4-isoxazolepropionic acid receptor
CaMKII: Ca^2+^/calmodulin-dependent kinase II
KIBRA: KIdney BRAin protein
K-ZAP: KIBRA-PKMζ antagonist peptide
LTP: long-term potentiation
PICK1: protein interacting with C-kinase 1
PKCι/λ: protein kinase C iota/lambda
PKCζ: protein kinase C zeta
PKMζ: protein kinase Mzeta
WWC1: WW and C2 Domain Containing protein 1

## Declarations

This study was performed in strict accordance with the recommendations in the Guide for the Care and Use of Laboratory Animals of the National Institutes of Health.

## References

[1] Crick F. Memory and molecular turnover. Nature. 1984;312(5990):101. 6504122 10.1038/312101a0

[2] Lisman JE. A mechanism for memory storage insensitive to molecular turnover: a bistable autophosphorylating kinase. Proc Natl Acad Sci U S A. 1985;82(9):3055–7. 2986148 10.1073/pnas.82.9.3055 PMC397705

[3] Sacktor TC. Fenton 2018 What does LTP tell us about the roles of CaMKII and PKMzeta in memory? Mol Brain 11 1 77. 30593289 10.1186/s13041-018-0420-5 PMC6309091

[4] Bayer KU, Giese KP. A revised view of the role of CaMKII in learning and memory. Nat Neurosci 2024. 10.1038/s41593-024-01809-x 39558039

[5] Sacktor TC. How does PKMζ maintain long-term memory? Nat Rev Neurosci. 2011;12(1):9–15. 21119699 10.1038/nrn2949

[6] Hernandez AI, Blace N, Crary JF, Serrano PA, Leitges M, Libien JM, Weinstein G, Tcherapanov A, Sacktor TC. Protein kinase Mζ synthesis from a brain mRNA encoding an independent protein kinase Cζ catalytic domain. Implications for the molecular mechanism of memory. J Biol Chem. 2003;278(41):40305–16. 12857744 10.1074/jbc.M307065200

[7] Hsieh C, Tsokas P, Grau-Perales A, Lesburgueres E, Bukai J, Khanna K, Chorny J, Chung A, Jou C, Burghardt NS, et al. Persistent increases of PKMzeta in memory-activated neurons trace LTP maintenance during spatial long-term memory storage. Eur J Neurosci. 2021;54(8):6795–814. 33540466 10.1111/ejn.15137 PMC8333175

[8] Han J, Grau-Perales A, Harris RM, Kao H-Y, Pal A, Alarcon JM, Sacktor TC, Martiniani S, Hofmann HA, Fenton AA. Persistently increased expression of PKMzeta and unbiased gene expression profiles identify hippocampal molecular traces of a long-term active place avoidance memory and ‘shadow’ proteins. Adv Sci. 2026, in press. 10.1002/advs.202521254 PMC13185853 41824523

[9] Zhang L, Yang S, Wennmann DO, Chen Y, Kremerskothen J, Dong J. KIBRA: In the brain and beyond. Cell Signal. 2014;26(7):1392–9. 24642126 10.1016/j.cellsig.2014.02.023 PMC4032603

[10] Tsokas P, Hsieh C, Flores-Obando RE, Bernabo M, Tcherepanov A, Hernandez AI, Thomas C, Bergold PJ, Cottrell JE, Kremerskothen J, et al. KIBRA anchoring the action of PKMzeta maintains the persistence of memory. Sci Adv. 2024;10(26):eadl0030. 38924398 10.1126/sciadv.adl0030 PMC11204205

[11] Buther K, Plaas C, Barnekow A, Kremerskothen J. KIBRA is a novel substrate for protein kinase Czeta. Biochem Biophys Res Commun. 2004;317(3):703–7. 15081397 10.1016/j.bbrc.2004.03.107

[12] Ferguson L, Hu J, Cai D, Chen S, Dunn TW, Pearce K, Glanzman DL, Schacher S, Sossin WS. Isoform specificity of PKMs during long-term facilitation in Aplysia is mediated through stabilization by KIBRA. J Neurosci. 2019;39(44):8632–44. 31537706 10.1523/JNEUROSCI.0943-19.2019 PMC6820206

[13] Shouval HZ, Hsieh C, Flores-Obando RE, Cano DA, Tracy TE, Sacktor TC. Maintenance of memory by negative feedback of synaptic protein elimination: modeling KIBRA-PKMzeta dynamics in LTP. Learn Mem. 2025;32:9–10. 10.1101/lm.054077.124 PMC12543028 41101994

[14] Vogt-Eisele A, Kruger C, Duning K, Weber D, Spoelgen R, Pitzer C, Plaas C, Eisenhardt G, Meyer A, Vogt G, et al. KIBRA (KIdney/BRAin protein) regulates learning and memory and stabilizes protein kinase mzeta. J Neurochem. 2013;128(5):686–700. 24117625 10.1111/jnc.12480 PMC3947452

[15] Shao X, Volk L. PICK1 links KIBRA and AMPA receptor subunit GluA2 in coiled-coil-driven supramolecular complexes. J Biol Chem. 2025;301(5):108397. 40074086 10.1016/j.jbc.2025.108397 PMC12136796

